# Bacterial Endophyte Colonization and Distribution within Plants

**DOI:** 10.3390/microorganisms5040077

**Published:** 2017-11-25

**Authors:** Shyam L. Kandel, Pierre M. Joubert, Sharon L. Doty

**Affiliations:** School of Environmental and Forest Sciences, College of the Environment, University of Washington, Seattle, WA 98195-2100, USA; Shyam.kandel@ars.usda.gov (S.L.K.); pierrj@uw.edu (P.M.J.)

**Keywords:** bacterial endophytes, colonization, microscopy, *Populus* endophytes

## Abstract

The plant endosphere contains a diverse group of microbial communities. There is general consensus that these microbial communities make significant contributions to plant health. Both recently adopted genomic approaches and classical microbiology techniques continue to develop the science of plant-microbe interactions. Endophytes are microbial symbionts residing within the plant for the majority of their life cycle without any detrimental impact to the host plant. The use of these natural symbionts offers an opportunity to maximize crop productivity while reducing the environmental impacts of agriculture. Endophytes promote plant growth through nitrogen fixation, phytohormone production, nutrient acquisition, and by conferring tolerance to abiotic and biotic stresses. Colonization by endophytes is crucial for providing these benefits to the host plant. Endophytic colonization refers to the entry, growth and multiplication of endophyte populations within the host plant. Lately, plant microbiome research has gained considerable attention but the mechanism allowing plants to recruit endophytes is largely unknown. This review summarizes currently available knowledge about endophytic colonization by bacteria in various plant species, and specifically discusses the colonization of maize plants by *Populus* endophytes.

## 1. Introduction

The term “endophyte” is derived from the Greek words “endon” meaning within, and “phyton” meaning plant. Previously, endophytes were defined as microorganisms such as bacteria and fungi that inhabit the plant endosphere during all or part of their life cycle without causing any apparent harm to the host plant [1,2]. However, the definition of endophytes has been revised multiple times by different authors [1,3,4]. More recently, Hardoim et al. [4] defined endophytes as microbes including bacteria, archaea, fungi, and protists that colonize the plant interior regardless of the outcome of the association. Conventionally, endophytes were isolated from surface sterilized plant tissue and cultivated in nutrient rich medium. In recent years, many endophytes have been identified through culture-independent approaches such as sequencing of the 16S rRNA gene, the internal transcribed spacer regions, ITS1 and ITS2, or through whole genome sequencing of endophyte communities [5,6,7,8].

Bacterial endophytes that are beneficial to plant growth and development are the focus of this review. They are found across many phyla, including the Proteobacteria, Actinobacteria, Firmicutes and Bacteroidetes [4,9,10,11]. Increased biomass and height in inoculated plants have been reported as a result of colonization by many endophytic genera such as *Azoarcus*, *Burkholderia*, *Gluconobacter*, *Klebsiella*, *Pantoea*, *Herbaspirillum*, *Rahnella*, and *Pseudomonas* [12,13,14,15,16,17,18,19]. Common characteristics of endophytes include the ability to synthesize plant hormones such as indole-3-acetic acid, solubilize phosphate, secrete siderophores, and confer plant tolerance to biotic and abiotic stresses [20,21,22,23]. Additionally, some bacterial endophytes carry genes necessary for biological nitrogen fixation (BNF), potentially enabling them to convert dinitrogen gas (N_2_) into usable forms of nitrogen such as ammonium and nitrate within the host plant [24,25].

Symbiotic N-fixation by rhizobia in legume plants or *Frankia* in actinorhizal plants, respectively, has been an active area of research for decades. However, the discovery of N-fixing bacterial endophytes in the non-nodulating plants such as sugarcane during the late 1980′s has expanded the area of BNF research [26,27]. Bacterial endophytes in several genera such as *Azoarcus*, *Burkholderia*, *Gluconobacter*, *Herbaspirillum*, *Klebsiella*, *Pantoea*, and *Rahnella* were found in many different plants, facilitating the growth of the host plant in nutrient-poor conditions [10,22,28,29]. N-fixation involves reducing the triple bonds of N_2_ molecules, which requires substantial amounts of energy. Given this energy demand, free-living N-fixers likely have relatively limited applications in agriculture compared to plant-associated N-fixers, which can overcome the energy requirement of N-fixation by deriving energy from the host plant [30]. Bacterial endophytes reside in the internal plant tissues which may be a favorable environment for N-fixation that minimizes competition with other microbes in the rhizosphere as well as possibly providing a microaerobic environment that is necessary for nitrogenase activity [31,32,33].

The use of bacterial endophytes in agriculture has immense potential to reduce the environmental impacts caused by chemical fertilizers, especially N fertilizers. Several studies have shown that a significant portion of N used in agriculture is lost to the environment. It is estimated that only 17 Tg N of every 100 Tg N used in global agriculture is utilized [34,35,36]. The N lost from farmlands eventually accumulates in lakes, rivers or marine systems causing excessive growth of algae, which has serious impacts on aquatic ecosystems. Greater atmospheric N, in the form of ammonia or ammonium, also coincides with areas of eutrophication in the downwind regions of farmland. Elevated concentrations of N in the form of ammonium, nitrate or nitric acid vapors in the atmosphere can reduce air quality, reduce visibility and impact plant growth [37,38]. In addition, microorganisms convert excess ammonium or nitrate in the soil into nitrous oxide, which is a potent greenhouse gas. The use of natural symbionts such as bacterial endophytes could reduce the need for fertilizer inputs in the growth of crop plants and potentially lead to making farming more environmentally sustainable in the future.

Bacterial endophyte strains promote plant growth by synthesizing phytohormones including indole-3-acetic acid (IAA), cytokinins and gibberellins or through regulating internal hormone levels in the plant body [4,39,40]. IAA produced by endophytes within plants increases the number of lateral and adventitious roots, facilitating access to nutrients, and improving root exudation, offering more resources for soil microbes to interact with roots [40,41]. Growth enhancement by increasing plant height and/or biomass has been reported in many studies when plants were inoculated with bacterial endophytes capable of producing IAA [39,42,43,44,45]. Furthermore, bacterial endophytes secrete siderophores and solubilize phosphorus in soil while initiating the symbiotic interactions with host plants [4,41]. Siderophores are organic compounds secreted by microorganisms and plants in iron limited conditions enabling them to chelate iron from the environment for microbial and plant cells to uptake [4,46]. Similarly, phosphorus-solubilizing bacteria can solubilize immobile phosphorus in soil, which is potentially available for plants to absorb, an important trait for plant growth promotion [47,48,49,50]. Many recent reviews highlighted the mechanisms and importance of phosphorus solubilizing microorganisms in agriculture [4,51].

Bacterial endophytes can confer resistance or tolerance to the host plant from biotic and abiotic stresses by releasing antimicrobial compounds, producing siderophores, competing for space and nutrients, and modulating the plant resistance response [39,52,53]. Some bacterial strains can relieve plant stress by blocking the pathway of ethylene synthesis in plants. These bacteria utilize 1-aminocyclopropane-1-carboxylate deaminase, which helps to reduce ethylene concentrations accumulated in response to different stresses in plants, otherwise lethal to plant health [54]. Endophytic strains of *Bacillus*, *Burkholderia*, *Enterobacter*, *Pseudomonas*, and *Serratia* were found to be effective in suppressing the growth of pathogenic microorganisms in in vivo and in vitro conditions [53,55,56,57]. Moreover, endophyte strains in the genera *Bacillus*, *Enterobacter*, *Pseudomonas*, *Azotobacter*, *Arthrobacter*, *Streptomyces*, and *Isoptericola* were successful in alleviating drought, heat, and salt stress in different crop plants. More importantly, symbiotic plants with these endophytes were not only capable of relieving the stress but also significantly increased plant biomass and height [58,59,60,61,62]. However, the mechanisms used by bacterial endophytes to mitigate abiotic stress remain unclear.

## 2. Recruitment of Bacterial Endophytes by Host Plants

The rhizosphere is defined as the soil-root interface where complex interactions take place between the plant and surrounding soil microorganisms [9,63]. It has been reported that plants can release significant amounts of photosynthates or exudates from its roots, which influence microbial communities in the rhizosphere. Root exudates including organic acids, amino acids, and proteins may be involved in recruiting bacterial endophytes from the rhizosphere [9,64,65]. Root exudates likely contain substrates that initiate early communication between host plants and bacterial endophytes, and consequently steer the colonization process. For example, evidence of the involvement of oxalate in the recruitment of the beneficial bacterial strain *Burkholderia phytofirmans* PsJNby host plants has been reported [66]. In this study, a *Burkholderia phytofirmans* strain defective in oxalate utilization was used to inoculate lupine and maize plants that secrete moderate and low levels of oxalate, respectively. The mutant was observed in significantly less numbers in both maize and lupine plants 3 days after inoculation as compared to the wild type strain. Interestingly, inoculation with both wildtype and mutant strains resulted in significant differences in colonization by the two strains in lupine but not in maize. Oxalate was also observed in *Brachypodium* root exudates, and high numbers of Proteobacteria were detected in the *Brachypodium* rhizosphere [64].

Moreover, bacterial quorum sensing compounds are likely involved in communication with the plant root and the subsequent colonization process. The importance of these compounds in the colonization and growth promotion of plants by endophytes is supported by a recent study that showed that a quorum sensing mutant of *Bukholderia phytofirmans* PsJN could no longer efficiently colonize *Arabidopsis thaliana* and did not promote its growth [67]. Plants are likely directly involved in quorum sensing as well, given that some plant extracts have been shown to have quorum quenching capabilities which could protect them against pathogens and some quorum sensing molecules have been shown to have direct plant growth promoting effects [68]. Additionally, several endophytes of *Populus deltoides* were found to have LuxR homologs hypothesized to be involved in responding to plant derived compounds [69]. This study also found that many of the surveyed endophyte genomes contained LuxR-LuxI type quorum sensing gene pairs pointing to their importance in the endophytic lifestyle. The importance of quorum sensing compounds for plant-microbe interactions has been reviewed in detail by Hartmann et al. [70].

The native soil composition and host plant genotype are also considered important in the recruitment of bacterial endophytes by the host plant. A detailed study of root endophytes of *Arabidopsis* plants grown in different soils concluded that soil type likely influences the composition of the bacterial endophyte community found in the host roots. This indicates that different soil types may be inhabited by variable bacterial populations that serve as the initial inocula [9]. In addition, Wagner et al. [71] showed that bacterial communities (epiphytic and endophytic) in *Boechera stricta*, a perennial wild mustard plant, are highly similar in both leaves and roots supporting the hypothesis that the communities are recruited from the soil. This study also showed that environmental conditions such as soil nutrition, moisture, temperature, and host genotype and age have a direct influence on root and leaf bacterial communities. Diverse bacterial communities were reported in grass species *Dactylis glomerata*, *Festuca rubra*, and *Lolium perenne* under different management regimes, such as fertilizer application and mowing frequencies, indicating that agronomic operations may influence bacterial endophyte recruitment in cultivated plants. Interestingly, in these grasses, the functional profile of the bacterial communities was not correlated with changes in community composition at the species-level, suggesting that selection of endophytes by the plant may be functionally driven rather than driven by phylogeny [11]. Furthermore, direct influence of crop genotype and N fertilizer application on the diversity of N-fixing (diazotrophic) endophytes was detected in maize and rice plants [72,73]. A detailed study of the root microbiome of *Arabidopsis* showed that only a narrow subset of rhizosphere communities was able to colonize and establish in the root endosphere [74]. Overall, molecular mechanisms by which plants select specific bacterial endophytes over others remain largely unknown [66,70].

## 3. Attachment of Bacterial Endophytes to the Host Plant Surface

The attachment or adhesion of bacterial cells to the plant surface is considered the first step of the colonization process. Bacteria in the vicinity of the plant roots most likely swim towards the roots, using chemotactic affinities for root exudates. This is followed by attachment to the root surface, which is likely important in getting access to potential entry sites at lateral root emergence areas or other openings caused by wounds or mechanical injuries. The exopolysaccharides (EPS) synthesized by bacterial cells may facilitate the attachment of bacterial cells onto the root surface and may be important in the early stages of endophytic colonization. The EPS produced by endophytic bacterium *Gluconacetobacter diazotrophicus* Pal5 was reported as an essential factor for rice root surface attachment and colonization [75]. A recent colonization study in rice plants using *G. diazotrophicus* Pal5 showed that bacterial cells were shielded from oxidative damage by exopolysaccharides, which may be crucial for colonization. Additionally, free radical concentrations *in planta* were decreased by the application of EPS. Colonization was reduced in an EPS knockout strain of *G. diazotrophicus*. Interestingly, this reduction in colonization was rescued by the addition of EPS produced by the wild type strain [76]. In another study, Balsanelli et al. analyzed the mutant strains of *Herbaspirillum seropedicae* that are deficient in EPS production and concluded that EPS is not required for plant colonization, which could potentially point to a variation in the genes required for colonization across different endophyte species [77]. The biology of bacterial EPS including its synthesis, chemistry and functions were reviewed elsewhere [78].

Bacterial structures such as flagella, fimbriae or cell surface polysaccharides are also likely involved in the attachment of bacteria to the plant surface. While studying colonization of maize plants by endophyte *H. seropedicae*, Balsanelli et al. reported that bacterial lipopolysaccharide (LPS) is necessary for attachment and subsequent endophytic colonization of plant roots [79]. Later, it was also demonstrated that binding of N-acetyl glucosamine of LPS with maize root lectins is required for bacterial attachment and subsequent colonization inside the roots [80]. Bacterial adherence and colonization of the root interior likely happen in close succession given how quickly colonization is observed in roots after inoculation with bacterial endophytes [81,82,83]. The process of adherence of *Rhizobia* on legume roots, plant pathogenic bacteria on plant leaf or root surfaces, and *Agrobacterium* on roots of the host plant has been thoroughly studied in the past [84,85,86]. However, the mechanisms by which bacterial endophytes attach on plant surfaces remain relatively unexplored [87].

## 4. Entry of Bacterial Endophytes into the Host Plant

Bacterial endophytes initially attach to the root surface also called rhizoplane, and explore the potential entry sites to access the internal plant tissues. Openings in the roots where root hairs or lateral roots emerge, as well as stomata, wounds and hydathodes in the shoots are considered the main entry points that endophytes use to enter the host plant [4]. Endophytic bacteria likely utilize these natural discontinuities in the plant body to access the internal plant tissues. Moreover, some bacterial endophytes may modify the plant cell wall by secreting cell wall cellulolytic enzymes such as cellulases, xylanases, pectinases, and endoglucanases, which facilitate bacterial entry and spread within the plant tissues [81,88,89]. One study supported this hypothesis by observing that the frequency of entry of an endoglucanase mutant of *Azoarcus* sp. BH72 into rice roots was decreased as compared to the wild type strain and the mutant was unable to spread to the aerial plant parts [88]. Many colonization studies suggested that natural cracks at the lateral root emergence site are the most common entry sites for endophytic bacteria [4,14,81]. Furthermore, some bacteria use root apex and root hairs as entry points followed by endophytic colonization in root cortex and vascular tissues [90,91].

## 5. Bacterial Niches inside the Host Plant

Bacterial endophytes most often occupy intercellular spaces in the plant, most likely because these areas have an abundance of carbohydrates, amino acids, and inorganic nutrients [4,12,27]. They likely exclusively colonize the intercellular spaces of various plant parts including roots, leaves, stems, flowers, and seeds [14,18,81,92,93,94]. Colonization can be localized at the tissue level or systemically throughout the plant body. In the early stages of endophytic colonization, endophytes are first observed in root hairs, and subsequently in the root cortex [83,90,95]. Inoculated *Burkholderia* sp. strain PsJN was observed in cortical cells, endodermis, and xylem vessels, and colonization was especially strong at primary and secondary roots and at the base of lateral roots and root tips. Interestingly, in this study, both intracellular and intercellular colonization was observed [81]. In maize plants, bacterial endophytic colonization was stronger in the lower stem compared to the stem closer to the shoot apex [96]. The mobility of bacterial cells accompanied by the synthesis of cellulolytic enzyme may help endophytes to spread to aerial plant parts including leaves and stems [12,25,81].

In leaves, bacterial endophytes have been observed in the intercellular spaces of mesophyll, and xylem tissues and substomatal areas. Using green fluorescent protein (GFP) labeling and β-glucuronidase (GUS) staining, *Burkholderia* sp. strain PsJN was observed in xylem and substomatal chambers of inoculated leaves of grape vine plants. Interestingly, bacterial cells leaving through the stomatal aperture were also observed in grapevine leaves [81]. The demand for nitrogen in the production of rubisco and other photosynthetic enzymes may suggest an important role for BNF by bacterial endophytes in the leaves. For example, studies have shown that diazotrophic endophytes *Klebsiella variicola* colonized the mesophyll cells of sugarcane leaves; *Herbaspirillum* sp. colonized young leaves and shoots of wild rice; *Herbaspirillum seropedicae* Z67 colonized leaf vein, mesophyll cells, and substomatal cavities of rice leaves; and *Serratia marcescens* colonized the leaf sheaths and leaf aerenchyma of rice plants [12,97,98,99]. Niches of indigenous bacterial endophytes in different sections of grapevine leaf pieces were found by florescence in situ hybridization (FISH) and confocal laser scanning microscopy. Bacterial microcolonies were observed in leaf veins, trichomes, and cut sections of leaf pieces. Colonization was strong in various layers of the leaf tissue [100].

One relatively new area of research that remains poorly studied is intracellular colonization of plant cells by endophytes. Endophytes are known to typically colonize the intercellular spaces of plants but several examples of intracellular colonization of plants by bacteria have been reported recently [101]. These examples include the presence of intracellular bacteria in shoot-tips of banana, shoot meristem of Scotch pine, seedling roots of switchgrass and in micro propagated peach palm [102,103,104,105]. While this area of research is relatively new and unexplored, several hypotheses exist as to the potential colonization pathway intracellular endophytes use. Root hairs offer a logical point of entry for these endophytes as many cases of intracellular plant-microbe interactions begin with colonization of the microbe through intracellular access to root hairs. This is the case in the very well-studied legume-rhizobium symbioses and is one method reported to be used by some endophytes [90,106]. The role of each symbiotic partner in intracellular colonization remains unclear. Endophytes may be capable of gaining access to the intracellular space directly by secreting cell wall degrading enzymes or through a phenomenon known as rhizophagy [104,107]. Rhizophagy is a recently observed process in which roots of certain plants actively bring microbes in the soil into their cells, possibly in order to digest them and acquire essential nutrients from them [108].

The advantages to this peculiar kind of endophytic colonization remain unclear. One possible hypothesis is linked to the observation that intracellular colonization by endophytes is associated with a bombardment of the colonizing endophytes by intracellular hydrogen peroxide. This allowed the authors to use a hydrogen peroxide stain to detect the intracellular bacteria but also points to a potential advantage of this interaction for the plant [104]. Briefly, increasing intracellular reactive oxygen species (ROS) concentrations in the plant could acclimate the plant to ROS stress, which could increase its tolerance to stressors linked to ROS stress such as drought, heat and salt stress [109]. Survival in the intracellular environment is likely a specific adaptation of the endophytes to this environment and could provide the endophyte with a niche with low competition. The specificity of this adaptation is supported by a change of shape of the intracellular endophytes of switchgrass to an L-form lacking a cell wall as well as the fact that many of these endophytes are not currently culturable [102,104]. While this phenomenon seems widespread, the difficulty of culturing intracellular endophytes makes them very difficult to study [102]. Classic microbiology methods relying on culturing the endophytes, including fluorescent tagging, may be difficult to implement in the study of these intracellular endophytes. It is possible that a stronger reliance on next generation sequencing, metagenomics and FISH may be necessary to further study the life cycle and ecology of these endophytes.

## 6. Bacterial Genes Involved in Plant Colonization

The production of ROS, mainly superoxide (O_2_^−^), hydrogen peroxide (H_2_O_2_), and hydroxyl radicals (OH^−^), are well understood as being an important immediate plant defense response in plant-microbe interactions [110,111,112]. During the plant-endophyte interaction, ROS-detoxification occurs early on, after entry of the endophyte into the plant. During the early stages of rice root colonization, endophytic diazotrophic bacterium *Gluconacetobacter diazotrophicus* expressed ROS-deactivating genes such as superoxide dismutase (SOD) and glutathione reductase (GR) in greater amounts. Furthermore, SOD and GR mutants of *G. diazotrophicus* could not colonize rice roots supporting the hypothesis that ROS-deactivating genes are important during the initial stages of colonization [113]. In addition, a gum gene cluster, *gum*D, in *G. diazotrophicus*, involved in EPS production, was shown to be required for biofilm formation and plant colonization. A genomic survey using comparative genomics of endophyte strains hypothesized that many genes involved in biofilm production, adhesion, and motility contribute to plant colonization and the endophytic life style within the host plant [6,114,115,116,117]. The metabolic adaptations required for root attachment, modification of the plant cell wall and life in the microaerobic environment within the plant was reported in the endophyte strain *Herbaspirillum seropedicae*. Increased gene expression of genes linked to N-fixation, auxin production and ABC transporters during interaction with the host plant was reported in this strain [87]. Many genes involved in bacterial chemotaxis and secretion systems were found in bacterial groups colonizing the *Brachypodium* rhizosphere and may be expressed during the colonization of roots by these endophytes [64].

## 7. Colonization Cycle of Bacterial Endophytes in the Host Plant

Bacterial endophytes are capable of colonizing different seed parts including the embryo. These endophytes likely mobilize and grow in the developing seedlings during germination and early seedling growth [118,119,120]. As seedlings emerge and plant growth begins, interactions between the roots and the soil microbiome commence. Plant exudates fuel microbial activities in the rhizosphere, which facilitate the attachment and entry of bacteria into the plant roots. Eventually, certain endophytes initiate colonization of tissues beyond the roots such as the stems and leaves, and ultimately throughout the plant endosphere. The colonization pattern and growth promoting characteristics of bacterial endophytes in different plant species are presented in Table 1. Some bacterial endophytes also colonize flowers and seeds, and most likely get transferred vertically from the maternal endophyte community into the offspring [93,120]. Additionally, a recent study showed that endophytes could colonize corresponding seeds after the flowers were inoculated. Moreover, endophytes passed on to seeds resumed endophytic activity after the seeds were planted [93,120,121].

**Figure 1:**
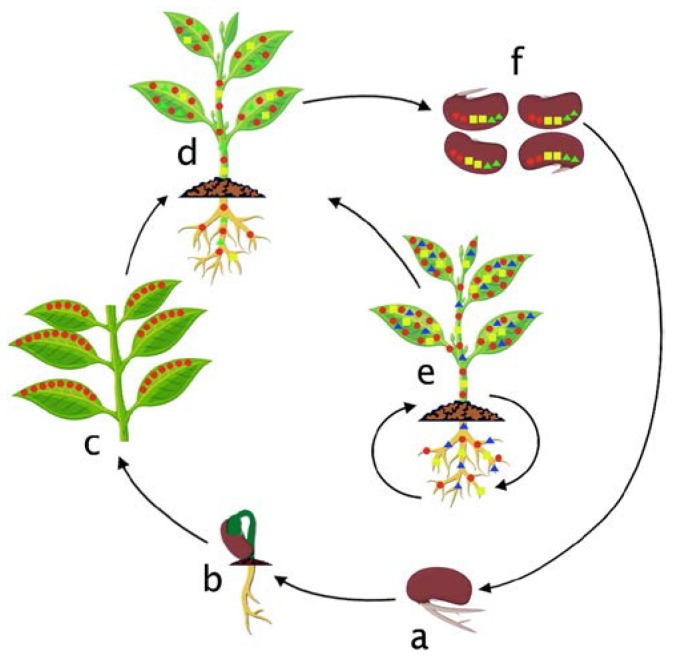
Hypothesized colonization cycle of bacterial endophytes in the host plant. (**a**) Mobilization of seed endophytes in germinating seedlings. (**b**) Recruitment of alien endophytes from the soil in developing seedlings. (**c**) Colonization by alien and inherited endophytes. (**d**) Whole plant colonization by various endophytes. (**e**) Variation of endophyte communities in the host plant in response to different biotic and abiotic stresses. (**f**) Vertical transfer of endophytes into seeds.

Because of their sessile lifestyle, plants are continuously challenged by different biotic and abiotic stresses including diseases, herbivory, heat, drought, and salinity. Endophytes likely manipulate their functional traits that allow them to interact with the host plant and respond rapidly to mitigate the consequences of adverse growth conditions [122,123]. The presence of distinct endophyte communities in different environmental conditions and different stages of the host life cycle indicates that specific functional groups of bacteria are likely to be active in response to a particular stress. In addition to vertically transmitted endophytes, “alien endophytes” (new cohorts of endophytes) accrue in the plant endosphere during plant growth. The “alien endophytes” can colonize various plant parts and incorporate new functional traits to the phytobiome through horizontal gene transfer with other microorganisms and can also eventually result in the loss of traits which may no longer be useful to the plant [124]. The hypothesized colonization cycle of bacterial endophytes in different growth stages of host plant is summarized in Figure 1.

## 8. Methods Used in Colonization Studies

Plating studies to determine the number of colony forming units (CFU) of endophytes within the plant in addition to microscopy based techniques used to visualize individual bacterial cells and/or microcolonies, as well as modern genomic sequencing-based approaches are all common techniques used to investigate the colonization of inoculated or indigenous bacterial endophytes in plants. They are briefly summarized here but have been extensively reviewed recently [14,18,92,100,123].

### 8.1. Cultivation Based Studies

In this method, selective or semi-selective culture medium is used to grow bacteria taken from plant extracts in order to determine the number of viable cells found in the plant tissue. The CFU count of bacterial endophytes from surface sterilized above ground and below ground tissues are used as an estimate of the internal populations of endophytes in the host plant [18,95,137,155]. The culturable population of bacterial endophytes can be quantified as the number of CFU per gram of root, shoot or leaf. Despite the ease and usefulness of this method, a portion of surface inhabiting epiphytes that are resistant to sterilizing agents such as ethanol or bleach can cause overestimation of endophyte counts. Additionally, this technique is only applicable to culturable bacterial endophytes. Recent studies based on genomic approaches suggest that a significant portion of bacterial communities is omitted by culture dependent approaches.

### 8.2. Microscopy Based Studies

Various types of microscopy such as bright-field microscopy, florescence microscopy, laser scanning confocal microscopy, and transmission electron microscopy have been commonly used to capture the colonization patterns of bacterial endophytes. The combination of microscopy with distinguishing fluorochromes or fluorescent dyes used to label or stain specific bacterial strains or bacterial communities allows the detection of endophytic colonization inside the plant tissues. FISH, GFP tagging, GUS staining, and fluorogenic dye staining are common techniques associated with microscopy to investigate the colonization of bacterial endophytes in plants [14,18,95,155]. In FISH, universal oligonucleotide probes targeting a conserved region of the 16S rRNA gene or species-specific probes are used to facilitate the observation of individual bacterial cells or microcolonies in the plant endosphere [95,99]. The use of broad host range plasmids containing constitutively expressed GUS or GFP genes are useful for tracking bacterial colonization inside the plants [63,89,104,137]. Bacterial endophytes tagged with GFP constitutively express the fluorescent proteins in situ, which allows entire bacterial cells to fluoresce in the presence of ultraviolet light or blue light, and oxygen [156,157]. In situ fluorescence of bacterial cells in plant tissue allows the localization and elucidation of the dynamics of colonization in different plant parts. The use of GFP tagged endophytes helps to assess the success of colonization, determine sites of bacterial entry, and investigate the microhabitat colonized inside the plant tissues [12]. In contrast to GUS staining, GFP is preferable for observing live cells. With GFP, plant tissues do not need to be fixed and no substrate or cofactor is necessary [158,159,160].

In many colonization studies, the combination of different techniques such as FISH, GFP labeling and GUS staining have been used [94,95,100]. By using a GFP gene fusion to a gene of interest, it is also possible to study and quantify the expression of a specific bacterial gene *in planta*. Egener et al. observed high levels of expression of nitrogenase in the rice root using GFP and immunogold labeling in the Kallar grass endophyte *Azoarcus* sp. BH72 [161]. Different microscopy based techniques can also be useful to study signaling pathways of plant endophyte interactions, mechanisms of host specificity, and, in general, the life style of endophytes within plants. Auto-fluorescence produced from the plant cell wall or organelles particularly in the leaf tissues may limit the use of these techniques but treating the specimens with low concentrations of bleaching agents may improve the image quality.

### 8.3. Genomics Based Studies

Recent advances in genetic tools and resources provide an important opportunity to improve our understanding of plant-endophyte interactions. Next generation sequencing technologies and bioinformatics tools allow the characterization of many endophyte communities from a variety of plant species [114,115,116,162]. Many recent studies have analyzed 16S rRNA gene sequences to study bacterial communities associated with the plant body [4,20,71,163,164]. The ubiquity of the 16S rRNA gene and its distinct evolutionary pattern allow its wide application in studying bacterial communities existing in different environments [165]. Due to the increasing accessibility of the technology, it is now becoming possible to analyze thousands of rRNA gene sequences of bacterial endophytes present in host plants. Genomics based studies have uncovered many novel bacterial communities in various plant parts. In these studies, the bacterial endophytes are described as operational taxonomic units based on sequence identity of the 16S rRNA gene or grouped into particular taxonomic units by phylogenetic analysis [9,71,74,164]. Modern techniques such as metagenomics and transcriptomics can provide information about the functional characteristics of endophyte species [166]. However, some bacterial traits are less conserved across phylogenetic lineages and differ in closely related strains of >98% identical in 16S rRNA sequence [124]. Further improvements in these techniques could possibly allow the identification of individual species or strains or a putative functional unit in a bacterial community that is critical for interaction with host plants and subsequent growth-promotion.

## 9. Poplar Endophytes and Their Colonization Efficiency in Crop Plants

To illustrate the methodology used in studying plant colonization by endophytes, we provide here an example of cross-species colonization of poplar tree endophytes with the monocot grass, maize. The Salicaceae (poplar and willow) endophytes have especially broad host ranges, and with the importance of the trees in environmental applications and bioenergy, and in genomics research as the first sequenced tree genome, poplar makes an excellent model system for the study of plant-microbe interactions. Poplar trees (*Populus* spp.) are early colonizers of nutrient poor habitats particularly wetlands, riparian areas, or other frequently disturbed areas. They can rapidly colonize open spaces made available after disturbances [167]. Because of their rapid and hardy growth, they are considered important plants for biomass production in the bioenergy industry. In addition, they provide various environmental services including conservation of soil, water, and biodiversity, and help to clean the contaminated sites by toxic chemicals [135,167,168]. Many endophyte strains are known to colonize and reside in poplar trees. The majority of the endophytic strains are members of the Alphaproteobacteria, Betaproteobacteria and Gammaproteobacteria classes but also include some yeast species. The majority of endophyte strains isolated from wild poplar in western Washington, USA, were identified as *Burkholderia*, *Curtobacterium*, *Rahnella*, *Pseudomonas*, *Acinetobacter*, *Pantoea*, *Rhodotorula*, and *Rhizobium* species [10,43,169]. Many of them can grow in N limited medium, possess a *nifH* gene, and are positive for the acetylene reduction assay, a common assay used to assess nitrogenase activity [10,43,57]. In addition, many of these strains produce substantial amounts of the plant growth hormone, indole-3-acetic acid [43,57]. Hacquard and Schadt recently reviewed the microbial communities of above ground and below ground tissues of *Populus* trees [170]. They highlighted the contribution to host plant health of microbial communities residing in the endosphere or in the phyllosphere.

Using the ^15^N dilution assay, it has been shown that endophytes in poplar plants contributed approximately 65% of the total N in leaves and increased plant biomass through biological N-fixation [152]. Khan et al. showed that inoculation of various crop plants with endophytes from poplar and willow trees resulted in earlier flowering and greater fruit yield in tomato and pepper, and higher biomass in maize, tomato, pepper, and squash in N limited conditions [171]. Additionally, sweet corn plants inoculated with endophytes showed increased plant biomass and improved photosynthetic capacity in leaves [19]. Furthermore, it has been shown that these endophytes colonized rice and maize plants effectively and resulted in greater biomass under nutrient limited conditions [172].

In this review, we present a novel example of a study of colonization by endophytes using a poplar endophyte. Here we provide further details on the colonization pattern of GFP-labeled strain, WP5*gfp* (*Rahnella* sp.) in two maize hybrids 29B17 and 14A91. Maize seedlings were germinated aseptically in the growth chamber after being surface sterilized. One-week-old seedlings were then inoculated by overnight co-cultivation with endophyte culture, and grown aseptically in the growth chamber in N free growth medium for 2 or 3 weeks. At the end of the experiments, inoculated seedlings were harvested, and rinsed multiple times with sterile water. Fresh root and shoot biomass were recorded and then used to determine colonization by CFU count and microscopy.

Fluorescent microscopy was used to observe the *in planta* population of WP5*gfp* in inoculated maize seedlings. Root systems including root hairs, lateral seminal roots, and leaf lamina were observed under the microscope to detect the colonization pattern of endophytes in the plant endosphere. The photographs were taken at 400 or 630 times magnification using transmission light or a GFP filter to visualize GFP fluorescence. For negative controls, mock-inoculated plant tissues were used. The endophytic population of WP5*gfp* was estimated in surface sterilized roots and shoots (stem and leaves) by cultivating bacterial cells in Mannitol Glutamate/Luria (MG/L) medium with 100 μg mL^−1^ of gentamycin and carbenicillin [173]. Dilution plates were incubated overnight at 30 °C, colonies were counted, and results were expressed as CFU per gram of plant biomass.

**Figure 2:**
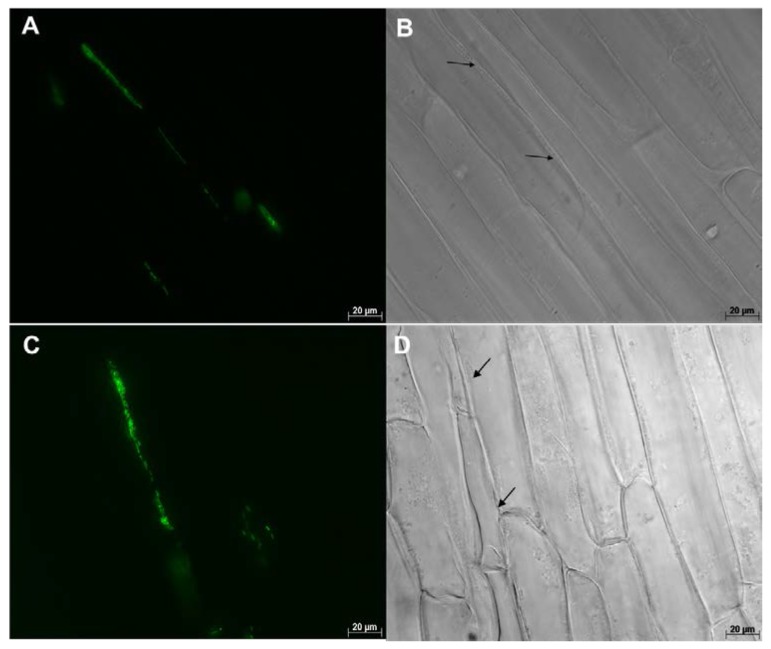
Maize (hybrid 29B17) roots colonized by WP5*gfp* visualized under 630× magnification. Image on the left (**A**,**C**) were taken with the GFP filter, and images on the right (**B**,**D**) were taken without the GFP filter. Groups of WP5*gfp* cells were observed in the intercellular spaces of cell layers in the longitudinal direction.

**Figure 3:**
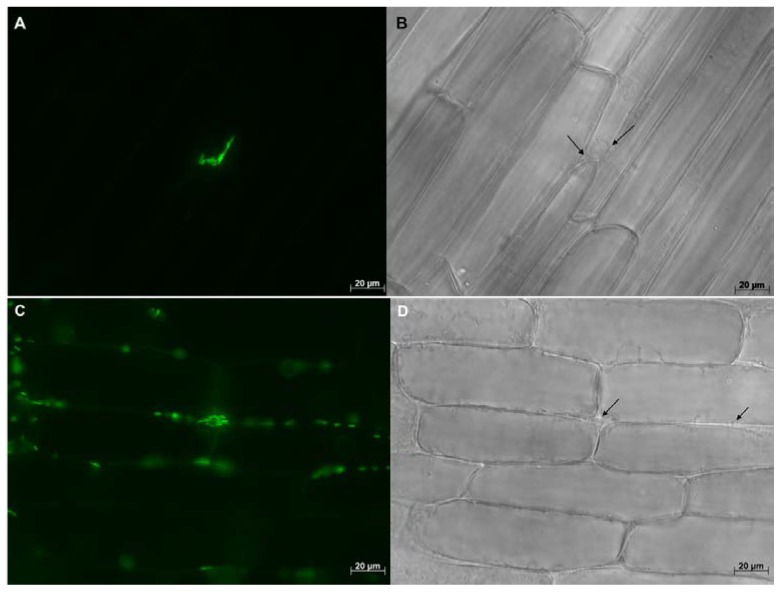
Maize (hybrids 14A91 and 29B17) roots were colonized by WP5*gfp* and visualized under 630× magnification. Image on the left (**A**,**C**) were taken with the GFP filter, and images on the right (**B**,**D**) were taken without the GFP filter. WP5*gfp* cells were observed in the middle lamella areas of the transverse wall between two adjacent cells.

**Figure 4:**
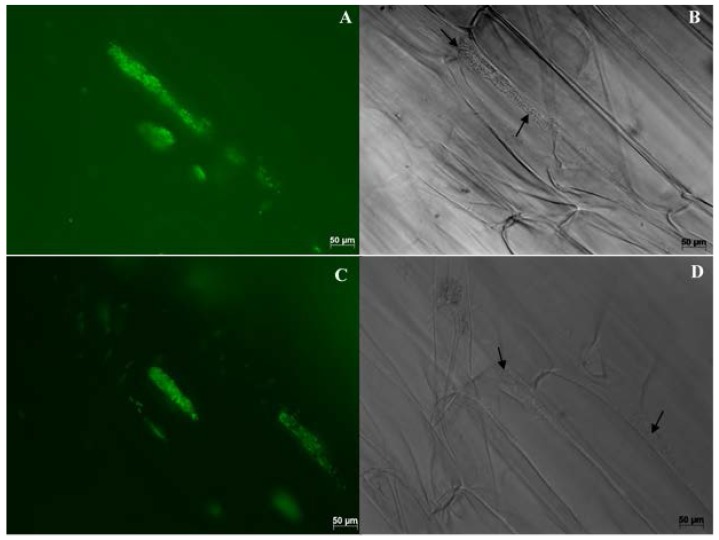
Maize (hybrid 29B17) radicle roots were colonized by WP5*gfp* and visualized under 630× magnification. Images on the left (**A**,**C**) were taken with the GFP filter, and images on the right (**B**,**D**) were taken without the GFP filter. Microcolonies of WP5*gfp* were observed along the plant cell wall areas between two adjacent cells.

**Figure 5:**
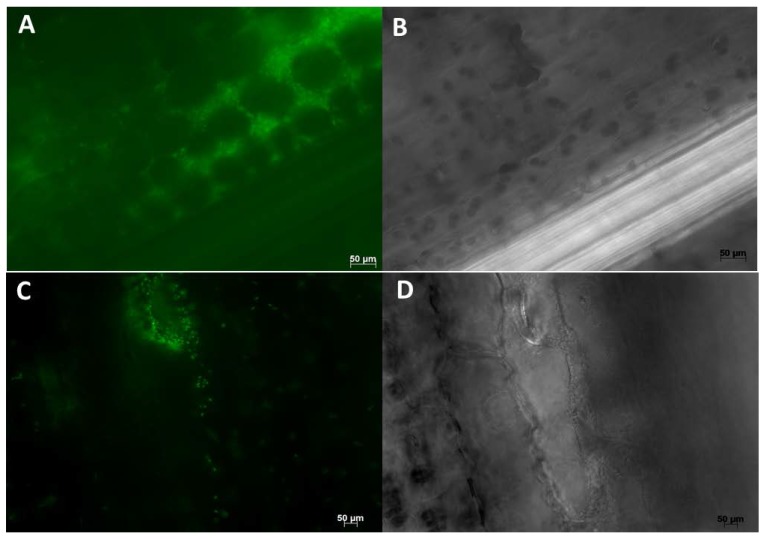
Maize (hybrid 29B17) leaves were colonized by WP5*gfp* visualized under 630× magnification. Image on the left (**A**,**C**) were taken with the GFP filter, and images on the right (**B**,**D**) were taken without the GFP filter. WP5*gfp* was observed in the intercellular spaces of cell layers and stomatal chambers.

Multiple samples were thoroughly checked under the microscope and nearly all inoculated plants were found to be colonized by WP5*gfp*. WP5*gfp* populations were observed repeatedly in elongation and differentiation zones of lateral seminal roots. They were often observed in between cells, in the intercellular spaces of cell layers in the longitudinal direction (Figure 2 and Figure 4), and in middle lamella areas of the transverse wall between two adjacent cells (Figure 3). They extensively used intercellular spaces and cell junctures as microhabitats for colonization in both roots and leaves. Bacterial cell growth adjoined to the plant cell wall was ubiquitous in all observed samples. In leaves, colonization was not detected in the midrib area but strong colonization was observed in the intercellular spaces of mesophyll cells (Figure 5A,B), and stomatal chambers in leaves (Figure 5C,D).

**Figure 6:**
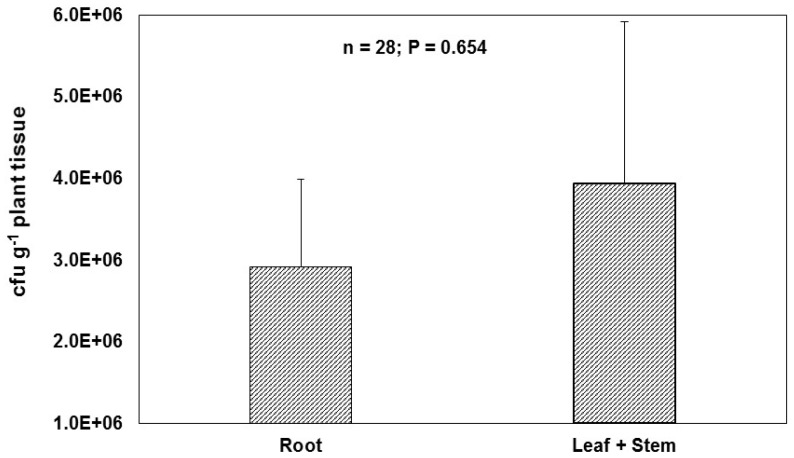
Quantification of CFUs of WP5*gfp* per gram of tissue in shoots, including leaves and stem, and roots. Errors bars represent standard error of the mean.

WP5*gfp* was recovered from surface sterilized maize root, leaf and stem samples. Higher WP5*gfp* CFU was observed in leaves and stems in contrast to root samples (Figure 6). Average WP5*gfp* CFU counts were 2.9 × 10^7^ per gram of roots, and 3.9 × 10^7^ per gram of leaves and stems. No colonies were observed in the mock-inoculated control plants. In another study, significantly higher numbers of CFUs were also observed in shoots than roots [12]. The bigger areas of leaves and stems may allow higher numbers of endophytes to colonize compared to the roots.

**Figure 7:**
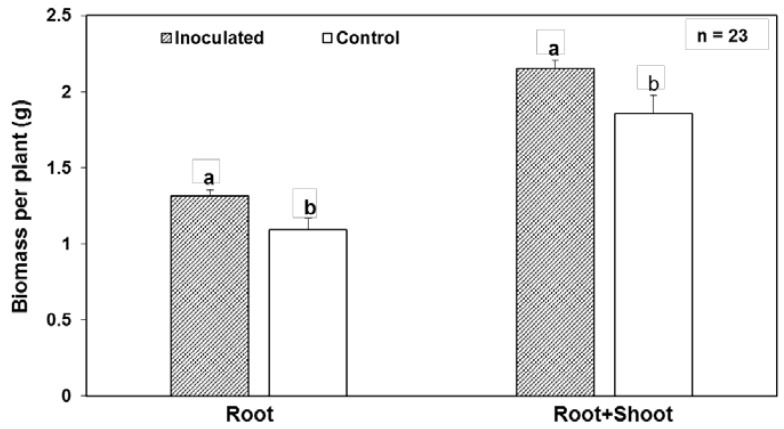
Root, and root and shoot biomass of WP5*gfp* inoculated and mock-inoculated control plants in maize hybrid 29B17. Error bars represent standard error of the mean. Histograms with different letters are statistically different at *p* < 0.05.

Fresh root biomass as well as combined root and shoot biomass (g plant^−1^) were significantly higher in inoculated plants as compared to the mock control groups (*p* = 0.011 and *p* = 0.021, respectively). Root weight was 20% higher in inoculated plants, and combined root and shoot weight was 16% higher (Figure 7). WP5*gfp* inoculated plants had greater root bulk and bigger overall plant stature as compared to mock inoculated plants.

As reported in other colonization studies, WP5*gfp* can colonize crop plants such as maize and rice beyond its native host plant poplar. Bacterial endophytes can colonize a variety of plants regardless of taxonomic isolation [4,22,174]. WP5*gfp* colonized roots, leaves, and stems with high populations and resulted in increased plant growth as compared to mock-inoculated control plants. WP5*gfp* probably promptly attached to the plant surface and entered into the internal tissues as soon as they were exposed to the maize plants, and subsequently multiplied in intercellular spaces, and xylem tissues. Since colonization was widespread and resulted in positive growth response in maize seedlings, WP5 is expected to be a suitable strain to use in the production of maize with reduced addition of synthetic fertilizers. Plant growth promoting activities of other endophyte strains in the *Rahnella* genus have been described in many native and inoculated host plants, which supports the results presented here [18,19,175].

## 10. Conclusions

The focus of this review is to summarize the colonization, from recruitment, attachment, and entry to the distribution patterns of bacterial endophytes in the plant endosphere. The rhizosphere serves as a hub for plant-endophyte communication during the early stages of the colonization process and likely facilitates access to the inside of the plant tissues through openings in the plant. Some bacterial endophytes have the potential to colonize all plant parts and interact beneficially with the host plant. As an illustration of the methods used to study colonization, a specific case of this interaction is presented using *Populus* endophytes and the colonization of maize plants. While effective colonization and increased biomass are demonstrated, many questions remain. The early signaling mechanisms and the exchange of signaling molecules between endophyte strains and host plants, as well as the temporal and spatial dimensions of the endophytic colonization process at the molecular level, have yet to be deciphered. Further studies on the molecular and biochemical basis of plant-endophyte interactions may uncover more details about the process of bacterial endophyte colonization.

## Figures and Tables

**Table 1:** Colonization of different plants by bacterial endophytes.

Endophyte Species	Native Host	Plant Colonized	Tissues Colonized	Effect on Plant	References
*Acetobacter diazotrophicus*	Sugarcane	Sugarcane	Stem	N/A	Dong et al., 1994 [27]
*Achromobacter* sp., and *Acinetobacter* sp.	*Poaceae* family (maize, wheat, pearl millet, sorghum and rice)	Wheat	Root	Growth enhancement	Patel et al., 2017 [125]
*Azoarcus* sp.	Kallar grass	Rice, and Kallar grass	Root, shoot	Growth enhancement	Hurek et al., 1994 [126]
*Azoarcus* sp.	Kallar grass	Rice	Root	N/A	Reinhold-Hurek et al., 2006 [88]
*Azospirillum* sp.	Maize	Maize	N/A	Growth enhancement	Riggs et al., 2001 [29]
*Bacillus megaterium*	Maize	Maize	Root, stem, leaf	N/A	Liu et al., 2006 [127]
*Bacillus pumilus*	Rice	Rice	Root	Growth enhancement	Bacilio-Jimenez et al., 2001 [128]
*Bacillus* sp.	Tomato	Wheat	N/A	Growth enhancement	Tian et al., 2017 [129]
*Bacillus* sp.	Maize	Maize	N/A	Growth enhancement	Riggs et al., 2001 [29]
*Bacillus subtilis*	Mulberry	Mulberry	Root, stem, leaf	Reduced bacterial wilt	Ji et al., 2008 [130]
*Burkholderia cepacia*	Maize	Maize	N/A	Growth enhancement	Riggs et al., 2001 [29]
*Burkholderia phytofirmans*	Onion	Grapevine	Root, stem, berry	N/A	Compant et al., 2008 [131]
*Burkholderia phytofirmans*	Onion	Grapevine	Root, stem, leaf	Growth enhancement	Compant et al., 2005 [81]
*Burkholderia phytofirmans*	Onion	Switchgrass	Root, leaf, sheath	Growth enhancement	Kim et al., 2012 [132]
*Burkholderia phytofirmans*	Onion	*Arabidopsis thaliana*	Root	Growth enhancement, increased chlorophyll content	Zuniga et al., 2013 [67]
*Burkholderia phytofirmans*	Onion	White lupin, and maize	Root, seed	N/A	Kost et al., 2014 [66]
*Burkholderia phytofirmans*	Onion	Maize	Root, stem, leaf	Growth enhancement, increased drought tolerance	Naveed et al., 2014 [60]
*Burkholderia* sp.	Tomato	Wheat	N/A	Growth enhancement	Tian et al., 2017 [129]
*Burkholderia vietnamiensis*	Poplar	Kentucky bluegrass	Root, shoot	Growth enhancement	Xin et al., 2009 [43]
*Burkholderia vietnamiensis*	Rice	Rice	Root	Growth enhancement	Govindarajan et al., 2008 [133]
*Burkholderia vietnamiensis*	Sugarcane	Sugarcane	Root	Growth enhancement, increased yield	Govindarajan et al., 2006 [134]
*Corynebacterium flavescens*	Rice	Rice	Root	Growth enhancement	Bacilio-Jimenez et al., 2001 [128]
*Enterobacter* sp.	Maize	Maize	Root, stem, leaf	Growth enhancement, increased drought tolerance	Naveed et al., 2014 [60]
*Enterobacter* sp.	Hybrid poplar (*Populus deltoides* × *P. nigra*)	Hybrid poplar	Root, leaf bud	Growth enhancement, reduced phytotoxicity of TCE, degradation of TCE	Doty et al., 2017 [135]
*Enterobacter* sp.	Tomato	Wheat	N/A	Growth enhancement	Tian et al., 2017 [129]
*Enterobacter* sp.	Maize	Maize	N/A	Growth enhancement	Riggs et al., 2001 [29]
*Gluconacetobacter diazotrophicus*	Sugarcane	Wheat, and sorghum	Root, shoot, stem, leaf	N/A	Luna et al., 2010 [136]
*Gluconacetobacter diazotrophicus*	Maize	Maize	N/A	Growth enhancement	Riggs et al., 2001 [29]
*Gluconacetobacter diazotrophicus*	Sugarcane	Rice	Root	N/A	Meneses et al., 2017 [76]
*Gluconacetobacter diazotrophicus*	Sugarcane	Sugarcane, and rice	Root, Shoot	N/A	Rouws et al., 2010 [137]
*Herbaspirillum seropedicae*	Maize	Maize	Root	N/A	Balsanelli et al., 2014 [77]
*Herbaspirillum seropedicae*	Maize	Maize	N/A	Growth enhancement	Riggs et al., 2001 [29]
*Herbaspirillum seropedicae*	Maize	Maize	Root	Increased rooting, change in gene expression	Amaral et al., 2014 [138]
*Herbaspirillum seropedicae*	Maize	Maize, wheat, rice and sorghum	Root, stem, leaf	N/A	Roncata-Maccari et al., 2003 [139]
*Herbaspirillum seropedicae*	Rice	Rice	Root, coleoptile, leaf	Growth enhancement	James et al., 2002 [99]
*Herbaspirillum seropedicae*	Sorghum	Maize	Root, leaf	N-fixation, change in metabolic profile	Brusamarello-Santos et al., 2017 [140]
*Herbaspirillum seropedicae*	Sorghum	Wheat	Root	Change in gene expression	Pankievicz et al., 2016 [87]
*Herbaspirillum* sp.	Rice (*Oryza officianalis*)	Rice (*Oryza* spp.)	Shoot, seed, leaf	Growth enhancement, N-fixation	Elbeltagy et al., 2001 [12]
*Klebsiella pneumoniae*	Maize	Alfalfa, *Arabidopsis*, wheat, and rice	Root, hypocotyl	N/A	Dong et al., 2003 [141]
*Klebsiella pneumoniae*	Maize	Wheat	Root	Growth enhancement, increased chlorophyll content, N-fixation	Iniguez et al. 2004 [14]
*Klebsiella pneumoniae*	Maize	Alfalfa	Root, hypocotyl	N/A	Dong et al., 2003 [141]
*Klebsiella* sp.	Maize	Maize	N/A	Growth enhancement	Riggs et al., 2001 [29]
*Microbacterium* sp.	Rape	Rape	Root	Growth enhancement, increased Pb uptake, root elongation,	Sheng et al., 2008 [142]
*Ochrobactrum* sp.	Rice	Rice	Root	N/A	Verma et al., 2004 [143]
*Pantoea agglomerans*	Maize	Maize	N/A	Growth enhancement	Riggs et al., 2001 [29]
*Pantoea agglomerans*	Rice	Rice	Root	N/A	Verma et al., 2001 [144]
*Pantoea* sp.	Rice	Rice	Root	N/A	Verma et al., 2004 [143]
*Pseudomonas fluorescences*	*Miscanthus*	Pea	N/A	Growth enhancement in phosphate limited conditions	Oteino et al. 2015 [48]
*Pseudomonas fluorescens*	Rape	Rape	Root	Growth enhancement, increased Pb uptake, root elongation,	Sheng et al., 2008 [142]
*Pseudomonas fluorescens*	Black nightshade	Black nightshade and tobacco	Root	Growth enhancement	Long et al., 2008 [145]
*Pseudomonas fluorescens*	Wheat	Tomato	Root	N/A	Duijff et al., 1997 [146]
*Pseudomonas putida*	Hybrid poplar	Willow	Root	Growth enhancement, reduced phytotoxicity of phenanthrene, degradation of phenanthrene	Khan et al., 2014 [147]
*Pseudomonas putida*	Potato	Potato	Root, stem	Growth enhancement, *Phytophthora infestans* suppression	Andreote et al., 2009 [148]
*Pseudomonas putida*	Poplar	Pea	Root, stem, leaf	Increased accumulation of and tolerance to 2,4-dichlorophenoxyacetic acid	Germaine et al., 2006 [149]
*Pseudomonas* sp.	Black nightshade	Black nightshade and tobacco	Root	Growth enhancement	Long et al., 2008 [145]
*Pseudomonas* sp.	Tomato	Wheat	N/A	Growth enhancement	Tian et al., 2017 [129]
*Pseudomonas* sp.	Poplar	Poplar	Root, stem, leaf	N/A	Germaine et al., 2004 [92]
*Pseudomonas thivervalensis*	Black nightshade	Black nightshade and tobacco	Root	Growth enhancement	Long et al., 2008 [145]
*Ralstonia* sp.	*Poaceae* family (maize, wheat, pearl millet, sorghum and rice)	Wheat	Root	Growth enhancement	Patel et al., 2017 [125]
*Rhanella aquatilis*	Sweet potato	Hybrid poplar	N/A	Increased rooting	Khan et al., 2009 [150]
*Rhizobium* sp.	Tomato	Wheat	N/A	Growth enhancement	Tian et al., 2017 [129]
*Rhizobium* sp.	*Poaceae* family (maize, wheat, pearl millet, sorghum and rice)	Wheat	Root	Growth enhancement	Patel et al., 2017 [125]
*Rhizobium* sp.	Maize	Maize	N/A	Growth enhancement	Riggs et al., 2001 [29]
*Serratia marcescens*	Rice	Rice	Root, stem, leaf	Growth enhancement	Gyaneshwar et al., 2001 [98]
*Staphylococcus* sp.	Tomato	Wheat	N/A	Growth enhancement	Tian et al., 2017 [129]
*Stenotrophomonas* sp.	Tomato	Wheat	N/A	Growth enhancement	Tian et al., 2017 [129]
Consortium (*Gluconacetobacter diazotrophicus*, *Herbaspirillum seropedicae*, *Herbaspirillum rubrisubalbicans*, *Azospirillum amazonense* and *Burkholderia* sp.)	Sugarcane	Sugarcane	Root, shoot	Growth enhancement, increased N content	Oliveira et al., 2002 [151]
Consortium (*Burkholderia vietnamiensis*, *Rhanella* sp., *Acinetobacter* sp., *Herbaspirillum* sp., *Pseudomonas putida*, *Sphingomonas* spp.	Poplar and willow	Sweet corn	Root, shoot	Growth enhancement, increased CO2 assimilation	Knoth et al., 2012 [19]
Consortium (*Burkholderia vietnamiensis*, *Rhanella* sp., *Enterobacter* sp., *Pseudomonas graminis*, *Acinetobacter* sp., *Herbaspirillum* sp., *Sphingomonas yanoikuyae*, *Pseudomonas putida*, *Sphingomonas*, *Sphingomonas yanoikuyae*)	Poplar and willow	Poplar and hybrid poplar	N/A	Growth enhancement	Knoth et al., 2014 [152]
Consortium (*Burkholderia vietnamiensis*, *Rhizobium tropici*, *Acinetobacter calcoaceticus*, *Rhanella* sp., *Burkholderia* sp., *Enterobacter asburiae*, *Sphingomonas yanoikuyae*, *Pseudomonas* sp., *Curtobacterium* sp.)	Poplar and willow	Hybrid poplar	N/A	Growth enhancement, increased drought tolerance	Khan et al., 2016 [45]
Consortium (*Burkholderia vietnamiensis*, *Rhizobium tropici*, *Acinetobacter calcoaceticus*, *Rhanella* sp., *Burkholderia* sp., *Sphingomonas yanoikuyae*, *Pseudomonas* sp., *Sphingomonas* sp.)	Poplar and willow	Rice	Root, shoot	Growth enhancement (N-limited conditions)	Kandel et al., 2015 [18]
Consortium (*Gluconacetobacter diazotrophicus*, *Herbaspirillum seropedicae*, *Herbaspirillum rubrisubalbicans*, *Azospirillum amazonense*, *Burkholderia tropica*)	Sugarcane	Sugarcane	Root	N/A	Oliveira et al., 2009 [153]
Consortium (*Pseudomonas* spp., *Paentbacillus* spp., *Sphingomonas azotifigens*)	Ryegrass and rice	Ryegrass	Root, stem, leaf	Growth enhancement, increased TFA	Castanheira et al., 2017 [95]
Consortium (*Rhizobium tropici* bv. *populus*, *Acinetobacter calcoaceticus*, *Rhanella* sp., *Burkholderia* sp., *Sphingomonas* spp.)	Poplar and willow	Douglas-fir	Root, needles	Growth enhancement (nutrient limited conditions)	Khan et al., 2015 [154]
